# Effect of Rifampin-Isoniazid-Containing Antituberculosis Therapy on Efavirenz Pharmacokinetics in HIV-Infected Children 3 to 14 Years Old

**DOI:** 10.1128/AAC.01657-18

**Published:** 2018-12-21

**Authors:** Awewura Kwara, Hongmei Yang, Sampson Antwi, Anthony Enimil, Fizza S. Gillani, Albert Dompreh, Antoinette Ortsin, Theresa Opoku, Dennis Bosomtwe, Anima Sarfo, Lubbe Wiesner, Jennifer Norman, Wael A. Alghamdi, Taimour Langaee, Charles A. Peloquin, Michael H. Court, David J. Greenblatt

**Affiliations:** a College of Medicine and Emerging Pathogens Institute, University of Florida, Gainesville, Florida, USA; b Department of Biostatistics and Computational Biology, University of Rochester School of Medicine and Dentistry, Rochester, New York, USA; c Directorate of Child Health, Komfo Anokye Teaching Hospital, Kumasi, Ghana; d Department of Child Health, School of Medical Sciences, Kwame Nkrumah University of Science and Technology, Kumasi, Ghana; e Deaprtment of Medicine, The Miriam Hospital, Providence, Rhode Island, USA; f Division of Clinical Pharmacology, Department of Medicine, University of Cape Town, Cape Town, South Africa; g Department of Pharmacotherapy and Translational Research, College of Pharmacy, University of Florida, Gainesville, Florida, USA; h Department of Clinical Pharmacy, College of Pharmacy, King Khalid University, Abha, Saudi Arabia; i Program in Individualized Medicine, Department of Veterinary Clinical Sciences, College of Veterinary Medicine, Washington State University, Pullman, Washington, USA; j Graduate Program in Pharmacology and Experimental Therapeutics, Sackler School of Graduate Biomedical Sciences and Department of Integrative Physiology and Pathobiology, Tufts University School of Medicine, Boston, Massachusetts, USA

**Keywords:** children, HIV, tuberculosis coinfection, efavirenz, antituberculosis therapy, drug-drug interactions, tuberculosis

## Abstract

We compared efavirenz pharmacokinetic (PK) parameters in children with tuberculosis (TB)/human immunodeficiency virus (HIV) coinfection on and off first-line antituberculosis therapy to that in HIV-infected children. Children 3 to 14 years old with HIV infection, with and without TB, were treated with standard efavirenz-based antiretroviral therapy without any efavirenz dose adjustments. The new World Health Organization-recommended antituberculosis drug dosages were used in the coinfected participants. Steady-state efavirenz concentrations after 4 weeks of antiretroviral therapy were measured using validated liquid chromatography with tandem mass spectrometry (LC-MS/MS) assays. Pharmacokinetic parameters were calculated using noncompartmental analysis. Between groups, PK parameters were compared by Wilcoxon rank-sum test and within group by signed-rank test. Of the 105 participants, 43 (41.0%) had TB coinfection. Children with TB/HIV coinfection compared to those with HIV infection were younger, had lower median weight-for-age *Z* score, and received a higher median efavirenz weight-adjusted dose. Geometric mean (GM) efavirenz peak concentration (*C*_max_), concentration at 12 h (*C*_12h_), *C*_min_, and total area under the curve from time 0 to 24 h (AUC_0–24h_) values were similar in children with HIV infection and those with TB/HIV coinfection during anti-TB therapy. Geometric mean efavirenz *C*_12h_, *C*_min_, and AUC_0–24h_ values were lower in TB/HIV-coinfected patients off anti-TB therapy than in the children with HIV infection or TB/HIV coinfection on anti-TB therapy. Efavirenz clearance was lower and AUC_0–24h_ was higher on than in patients off anti-TB therapy. Reduced efavirenz clearance by first-line anti-TB therapy at the population level led to similar PK parameters in HIV-infected children with and without TB coinfection. Our findings do not support modification of efavirenz weight-band dosing guidelines based on TB coinfection status in children. (The study was registered with ClinicalTrials.gov under registration number NCT01704144.)

## Abstract (precis)

We compared efavirenz pharmacokinetic (PK) parameters in children with tuberculosis (TB)/human immunodeficiency virus (HIV) coinfection on and off first-line antituberculosis therapy to that in HIV-infected children. Children 3 to 14 years old with HIV infection, with and without TB, were treated with standard efavirenz-based antiretroviral therapy without any efavirenz dose adjustments.

## INTRODUCTION

Tuberculosis (TB) is a common cause of morbidity and mortality in children with human immunodeficiency virus (HIV) infection. Children with HIV infection have an up to 20-fold increased incidence of TB (1, 2), and TB/HIV-coinfected children have an up to 6-fold greater risk of dying from TB than those with TB only (3, 4). The high TB mortality in TB/HIV-coinfected children is in part due to suboptimal response to drug therapy and high TB relapse rates due to ongoing immunosuppression (5, 6). In addition, suboptimal exposure of antiretroviral drug caused by drug-drug interactions (DDIs) during concurrent therapy may contribute to the high mortality rates in TB/HIV-coinfected children. Efavirenz-based antiretroviral therapy (ART) is preferred in HIV-infected children aged 3 years and older with and without TB coinfection (7). However, whether efavirenz dose should be adjusted during coadministration with anti-TB therapy in children is not well established, and current practice is based on extrapolation from evidence from adult studies (8).

Efavirenz is cleared primarily via metabolism by cytochrome P450 (CYP) 2B6 (8-hydroxylation), as well as by CYP2A6 (7-hydroxylation) and UDP-glucuronosyltransferase (UGT) 2B7 (direct *N*-glucuronidation) (9–12). First-line anti-TB regimen consists of a combination of rifampin, isoniazid, pyrazinamide, and ethambutol for 2 months and then rifampin and isoniazid for 4 months. Reductions in plasma concentrations of efavirenz when coadministered with anti-TB therapy are often attributed to the induction of drug clearance pathways by rifampin (13). However, isoniazid is a known mechanism-based inhibitor of CYP1A2, CYP2A6, CYP2C9, CYP2C19, and CYP3A4 enzymes (14–16) and contributes significantly to DDIs (16). In 2010, the World Health Organization (WHO) recommended increased dosages of the first-line TB drugs for children (17, 18), but whether the increased rifampin and isoniazid dosages adversely affect the nature or magnitude of the DDIs with efavirenz is unknown. In this study, we examined efavirenz pharmacokinetic (PK) parameters in Ghanaian HIV-infected children with and without TB coinfection who were treated with the same weight-band efavirenz dosing. In addition, we examined the effect of anti-TB therapy on efavirenz pharmacokinetics in the TB/HIV-coinfected children by comparing PK parameters on and off anti-TB therapy.

## RESULTS

### Study population.

During the study period, 144 HIV-infected children were enrolled, of whom 10 withdrew from the study or were lost to follow-up prior to PK sampling. Twenty-nine participants were excluded for the following reasons: 12 had very low concentrations throughout the sampling period suspicious for poor medication adherence, 10 had two efavirenz peak concentrations, and seven had a delayed peak at either 12 or 24 h post dose. The demographic and clinical characteristics of the 105 participants included in the study are shown in Table 1. The baseline characteristics between the two groups were similar, except that patients in the TB/HIV-coinfected group were significantly more likely to be younger, have lower body weight for age, and to have received a higher efavirenz dose in milligrams per kilogram. Of the 43 children with TB/HIV coinfection, 39 (90.7%) had pulmonary TB, and the median isoniazid and rifampin dosages were 10 mg/kg and 15 mg/kg, respectively.

**TABLE 1:** Baseline characteristics of study participants

Characteristic^*a*^	All (*n* =105)	HIV (*n* = 62)	TB/HIV (*n* = 43)	*P* value
Median (IQR) age (yrs)	7.0 (5.0 to 10.0)	8.5 (5.4 to 10.8)	6.3 (4.4 to 9.0)	0.027
Median (IQR) body wt (kg)	17.0 (13.5 to 23.2)	20.4 (15.0 to 25.2)	15.0 (13.0 to 19.6)	0.002
Median (IQR) ht (cm)	111.0 (95.0 to 125.0)	119.5 (98.0 to 130.0)	103.0 (93.0 to 120.0)	0.022
Age range (yrs)				0.039
3 to <5	29 (27.6)	13 (21.0)	16 (37.2)	
5 to <10	40 (38.1)	22 (35.5)	18 (41.9)	
10 to 14	36 (34.3)	27 (43.5)	9 (20.9)	
Sex				0.689
Male	60 (57.1)	34 (54.8)	26 (60.5)	
Female	45 (42.9)	28 (45.2)	17 (39.5)	
*CYP2B6* 516G→T genotype (*n* = 101)				0.492
GG	21 (20.8)	12 (20.0)	9 (22.0)	
GT	54 (53.5)	30 (50.0)	24 (58.5)	
TT	26 (25.7)	18 (30.0)	8 (19.5)	
Nutritional status (median [IQR])				
Weight-for-age *Z* score (*n* = 79)	–2.3 (–3.0 to –1.1)	–1.8 (–2.8 to –0.9)	–2.5 (–3.0 to –1.7)	0.043
Height-for-age *Z* score	–2.7 (–3.3 to –2.0)	–2.6 (–3.3 to –1.5)	–2.8 (–3.6 to –2.2)	0.249
BMI-for-age *Z* score	–0.5 (–1.8 to 0.3)	–0.4 (–1.5 to 0.2)	–0.7 (–2.2 to 0.5)	0.446
Median (IQR) efavirenz dose (mg/kg)	15.0 (13.7 to 16.9)	14.3 (13.2 to 16.2)	15.8 (15.0 to 18.8)	<0.001
Laboratory results (median [IQR])				
White blood cell count (×10^9^/liter) (*n* = 59)	6.4 (4.4 to 9.4)	6.2 (4.3 to 8.8)	7.2 (4.6 to 9.8)	0.474
Absolute neutrophil count (cell/) (*n* = 50)	2.2 (1.4 to 3.1)	2.4 (1.6 to 3.1)	1.7 (1.3 to 3.1)	0.458
Hemoglobin (g/dl) (*n* = 59)	10.3 (9.3 to 11.1)	10.4 (9.6 to 11.1)	10.0 (8.9 to 11.1)	0.202
Hematocrit (%) (*n* = 59)	30.3 (28.1 to 33.2)	30.3 (28.2 to 33.3)	30.4 (27.0 to 33.0)	0.365
Platelets (×10^9^/liter) (*n* = 58)	307.5 (217.0 to 422.0)	307.5 (230.0 to 416.0)	315.5 (209.5 to 483.0)	0.877
Blood urea nitrogen (mmol/liter) (*n* = 62)	2.6 (1.8 to 3.2)	2.7 (1.8 to 3.1)	2.5 (1.6 to 3.3)	0.578
Serum creatinine (µmol/liter) (*n* = 65)	35.0 (27.0 to 48.0)	34.0 (26.0 to 48.0)	35.5 (29.5 to 49.5)	0.474
Calculated GFR (ml/min/1.73 m^2^) (*n* = 65)	110.6 (88.9 to142.8)	115.6 (99.2 to 153.9)	100.9 (75.0 to 127.6)	0.046
Aspartate transferase (U/liter) (*n* = 72)	37.7 (28.5 to 49.0)	36.0 (25.0 to 45.7)	45.0 (31.0 to 57.2)	0.033
Alanine transferase (U/liter) (*n* = 70)	20.1 (15.0 to 33.0)	19.0 (14.4 to 26.0)	26.0 (16.2 to 42.5)	0.076
Alkaline phosphatase (U/liter) (*n* = 69)	330.0 (189.0 to 529.0)	331.0 (189.0 to 535.0)	330.0 (149.0 to 501.0)	0.486
Total bilirubin (µmol/liter) (*n* = 72)	5.0 (3.1 to 8.0)	5.0 (3.7 to 7.0)	6.5 (3.0 to 11.0)	0.473
Albumin (g/liter) (*n* = 70)	38.5 (34.0 to 42.7)	40.0 (35.0 to 42.8)	36.0 (28.5 to 41.0)	0.055
Median (IQR) HIV-related laboratory tests				
CD4 cell count (cells/µl) (*n* = 74)	397.5 (169.0 to 648.0)	467.0 (179.0 to 694.0)	283.0 (107.0 to 588.0)	0.277
CD4 percent (%) (*n* = 64)	14.0 (7.5 to 18.50	15.0 (9.0 to 22.0)	11.0 (6.0 to 18.0)	0.334
Log_10_ HIV-1 RNA (*n* = 62)	5.1 (4.4 to 5.8)	4.9 (4.3 to 5.4)	5.7 (4.8 to 6.0)	0.051
Nucleoside backbone (*n* = 104)^*b*^				0.257
Zidovudine + lamivudine	77 (73.3)	48 (77.4)	29 (67.4)	
Abacavir + lamivudine	27 (25.7)	13 (21.0)	14 (32.6)	
Median (IQR) drug dosages (mg/kg)				
Isoniazid			10.0 (9.0 to 11.7)	
Rifampin			15.0 (13.7 to 16.8)	
Pyrazinamide			25.0 (22.6 to 29.6)	
Ethambutol			16.7 (15.2 to 20.0)	

a Numbers (percent) are reported for categorical data. IQR, interquartile range; BMI, body mass index.

b One child received tenofovir disoproxil fumarate + lamivudine.

### Effect of anti-TB therapy on efavirenz pharmacokinetics.

**FIG 1:**
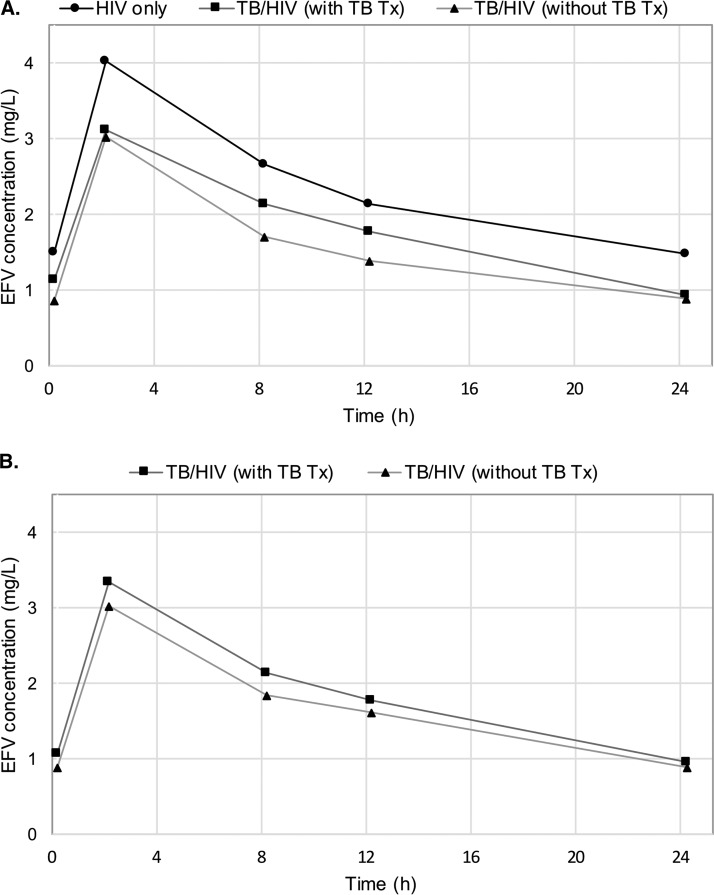
Median efavirenz plasma concentration plotted by sampling time after dosing. Panel A shows the plots for all participants in each treatment group, and panel B shows plots for TB/HIV-coinfected children who had paired samples on and off antituberculosis therapy.

The median efavirenz plasma concentration-time profiles in the HIV-infected children, without (*n* = 62) and with TB coinfection, on anti-TB therapy (*n* = 43) and off anti-TB therapy (*n* = 40) are higher in the children with HIV infection and lowest in those with TB/HIV coinfection off anti-TB therapy (Fig. 1A). In the 32-TB/HIV-coinfected children who had paired samples, the median concentrations of efavirenz were lower off compared to those of children on anti-TB therapy (Fig. 1B).

The geometric means (GM) of efavirenz peak concentration (*C*_max_), concentration at 12 h postdose (*C*_12h_), minimum concentration (*C*_min_), total area under the curve from time 0 to 24 h (AUC_0–24h_), and apparent oral clearance (CL/*F*) values in the children with TB/HIV coinfection on anti-TB therapy were similar to those in children with HIV infection (Table 2). There was also no significant difference in median PK parameter values between the children with TB/HIV coinfection on anti-TB and those with HIV infection (Table S1). We also compared PK parameters in the children with HIV infection and those with TB/HIV coinfection after stopping anti-TB therapy for at least 4 weeks. The GM of efavirenz *C*_12h_, *C*_min_, and AUC_0–24h_ were significantly lower, and GM CL/*F* was significantly higher, in the children with TB/HIV coinfection off anti-TB therapy compared to that in those with HIV infection alone (Table 2). The proportion of children in each group with efavirenz *C*_12h_ and *C*_min_ values outside the proposed therapeutic range of 1 to 4 mg/liter in adults (19), and the lower threshold of C_12h_ of 1.12 mg/liter, *C*_min_ of 0.65 mg/liter and AUC_0–24h_ of 28 mg*h/liter for poor virologic response in children (20) were similar, except that TB/HIV-coinfected children were significantly more likely to have an efavirenz *C*_min_ of <1 mg/liter than those with HIV infection (34.9% versus 14.5%, *P* = 0.019) (Table S2).

**TABLE 2:** Efavirenz pharmacokinetic parameter in HIV-infected children with and without tuberculosis (TB) coinfection expressed as geometric mean and 95% confidence interval

Parameter^*c*^	TB/HIV^*d*^					
	All (*n* = 105)	HIV (*n* = 62)	On ATT (*n* = 43)	Off ATT (*n* = 40)	*P* value^*a*^	*P* value^*b*^
*T*_max_ (h)	2.8 (2.5 – 3.2)	2.7 (2.3 – 3.1)	3.0 (2.5 – 3.7)	2.4 (2.1 – 2.8)	0.342	0.302
*C*_max_ (mg/liter)	4.0 (3.6 – 4.5)	4.2 (93.6 – 4.8)	3.9 (3.2 – 4.6)	3.3 (2.7 – 4.1)	0.532	0.060
*C*_12h_ (mg/liter)	2.3 (2.0 – 2.6)	2.3 (1.9 – 2.7)	2.2 (1.7 – 2.8)	1.6 (1.3 – 2.1)	0.727	0.017
*C*_min_ (mg/liter)	1.7 (1.4 – 2.1)	1.9 (1.6 – 2.3)	1.5 (1.0 – 2.1)	1.0 (0.7 – 1.5)	0.230	0.005
AUC_0–24h_ (mg · h/liter)	59.0 (51.9 – 67.2)	60.9 (51.8 – 71.5)	56.47 (45.3 – 70.5)	45.0 (35.8 – 56.6)	0.575	0.028
CL/*F* (liter/h)	4.7 (4.1 – 5.3)	4.7 (4.0 – 5.5)	4.7 (3.7 – 5.8)	6.2 (4.9 – 7.8)	0.942	0.044
*V*/*F* (liter)	141.5 (123.1 – 162.6)	146.3 (125.4 – 170.6)	134.8 (103.4 – 175.8)	175.2 (140.0 – 219.3)	0.595	0.171
*t*_1/2_ (h)	21.0 (18.1 – 24.3)	21.6 (18.8 – 24.8)	20.1 (14.8 – 27.3)	19.2 (16.0 – 23.0)	0.669	0.297

a *P* value for HIV versus TB/HIV on TB therapy.

b *P* value for HIV versus TB/HIV off TB therapy.

c AUC_0–24h_, total area under the curve from time 0 to 24 h; *C*_12h_, concentration at 12 h postdose; CL/*F*, apparent oral clearance; *C*_max_, peak concentration; *C*_min_, minimum concentration; *t*_1/2_, half-life; *T*_max_, time to *C*_max_; *V*/*F*, apparent volume of distribution.

d ATT, antituberculosis therapy.

**FIG 2:**
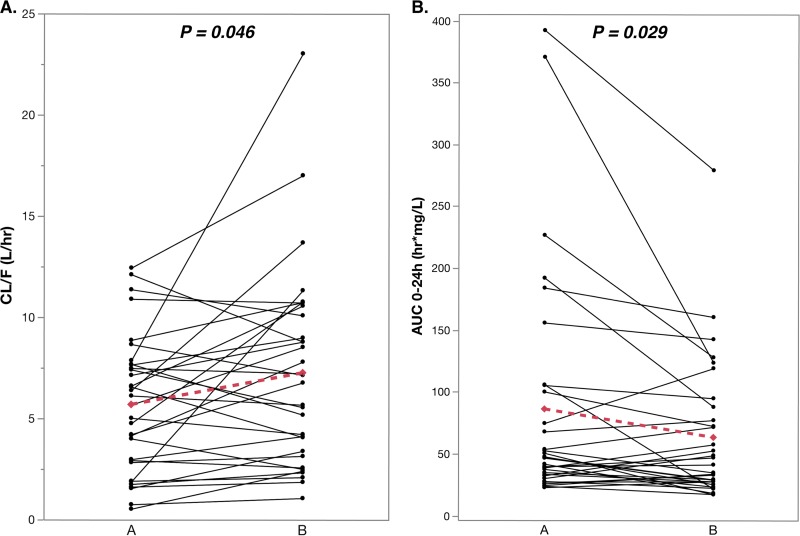
Efavirenz AUC_0–24h_ and CL/*F* on (A) and off (B) antituberculosis therapy in 32 TB/HIV-coinfected children with paired samples. The Fisher exact paired *t* test *P* value for mean change in PK parameters between the two periods (dotted line) is reported.

**FIG 3:**
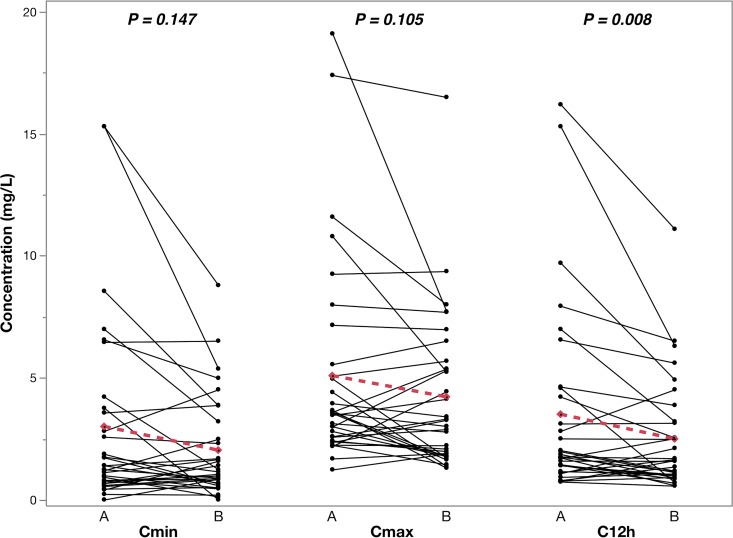
Efavirenz *C*_min_, *C*_max_, and *C*_12h_ values on (A) and off (B) antituberculosis therapy in 32 TB/HIV-coinfected children with paired samples. The Fisher exact paired *t* test *P* value for mean change in PK parameters between the two periods (dotted line) is reported.

Among the 32 TB/HIV-coinfected patients with paired samples on and off anti-TB therapy, the GM of efavirenz for *C*_max_, *C*_12h_, and AUC_0–24h_ were significantly lower, and CL/*F* was significantly higher, after stopping than during anti-TB treatment (Table 3). The pairwise analysis also showed a significant increase in mean efavirenz CL/*F* and a decrease in mean efavirenz AUC_0–24h_ between the periods on and off anti-TB therapy (Fig. 2). There was a decrease in mean *C*_min_, *C*_max_, and *C*_12h_ values off compared to on anti-TB therapy, but the changes were not significant except for that in *C*_12h_ (Fig. 3).

**TABLE 3:** Geometric means and 95% confidence intervals of efavirenz pharmacokinetic parameters on (PK1) and off (PK2) antituberculosis therapy and ratio of geometric means of PK1/PK2 in 32 HIV/TB coinfected children with paired samples^*a*^

Parameter^*d*^	PK1 GM (95% CI)^*b*^	PK2 GM (95% CI)	*P* value^*c*^	RGM of PK1/PK2 (90% CI)
*T*_max_ (h)	2.89 (2.31–3.60)	2.52 (2.09–3.05)	0.350	1.16 (0.89–1.49)
*C*_max_ (mg/liter)	4.01 (3.18–5.08)	3.37 (2.65–4.27)	0.048	1.19 (1.03–1.38)
*C*_12h_ (mg/liter)	2.31 (1.69–3.16)	1.82 (1.37–2.41)	0.018	1.27 (1.08–1.50)
*C*_min_ (mg/liter)	1.50 (0.93–2.41)	1.19 (0.76–1.84)	0.332	1.26 (0.85–1.89)
AUC_0-24h_ (mg · h/liter)	59.19 (44.29–79.11)	47.54 (36.44–62.02)	0.019	1.25 (1.07–1.45)
CL/*F* (liter/h)	4.49 (3.37–5.97)	5.79 (4.47–7.51)	0.011	0.77 (0.66–0.91)
*V*/*F* (liter)	135.21 (100.43–182.04)	162.07 (125.41–209.45)	0.208	0.83 (0.66–1.06)
*t*_1/2_ (h)	20.89 (14.42–30.25)	19.4 (15.77–23.86)	0.655	1.08 (0.82–1.42)

a PK1, PK on anti-TB therapy; PK2, PK off anti-TB therapy.

b GM, geometric mean; RGM, ratio of geometric mean; 95% CI, 95% confidence interval.

c *P* value comparing geometric means between PK1 and PK2.

d AUC_0–24h_, total area under the curve from time 0 to 24 h; *C*_12h_, concentration at 12 h post dose; *C*_max_, peak concentration; *C*_min_, minimum concentration; CL/*F*, apparent oral clearance; GM, geometric mean; *t*_1/2_ = half-life; *T*_max_, time to *C*_max_; *V*/*F*, apparent volume of distribution.

### Multivariate analysis of efavirenz pharmacokinetics.

In the multivariate model of predictors of efavirenz pharmacokinetics in the combined population, increased efavirenz *C*_max_, *C*_12h_, *C*_min_, and AUC_0–24h_ values were associated with younger age, larger efavirenz dose in milligrams, and *CYP2B6* 516 TT genotype status, while decreased CL/*F* was associated with younger age and *CYP2B6* 516 TT genotype status (Table 4). Other factors, such as TB coinfection status, sex, baseline weight, and height, were not associated with efavirenz PK parameters in the model.

**TABLE 4:** Multivariate analysis of the association of patient factors with efavirenz pharmacokinetic parameters in HIV-infected children with and without tuberculosis

PK parameter^*a*^	Predictor	Estimate	SE	Standardized estimate	*P* value
*C*_max_	Age (yrs)	−0.052	0.017	−0.346	0.004
	Dose (mg)	0.003	0.001	0.518	<0.001
	*CYP2B6* 516TT	0.576	0.080	0.544	<0.001
*C*_12h_	Age (yrs)	−0.048	0.019	−0.279	0.014
	Dose (mg)	0.003	0.001	0.366	0.001
	*CYP2B6* 516TT	0.774	0.088	0.637	<0.001
*C*_min_	Age (yrs)	−0.034	0.021	−0.177	0.121
	Dose (mg)	0.002	0.001	0.280	0.015
	*CYP2B6* 516TT	0.873	0.098	0.652	<0.001
AUC_0–24h_	Age (yrs)	−0.059	0.024	−0.274	0.016
	Dose (mg)	0.004	0.001	0.403	<0.001
	*CYP2B6* 516TT	0.957	0.109	0.630	<0.001
CL/*F*	Age (yrs)	0.042	0.012	0.260	<0.001
	*CYP2B6* 516TT	−0.739	0.083	−0.640	<0.001

a *C*_max_, peak concentration; *C*_12h_, concentration at 12 h postdose; *C*_min_, minimum concentration; AUC_0–24h_, total area under the curve from time 0 to 24 h; CL/*F*, apparent oral clearance. TB coinfection status, sex, weight, and height were not associated with efavirenz pharmacokinetic parameters in the multivariate model.

### Clinical outcome and virologic response by TB coinfection status.

Of the 134 children who initiated efavirenz-based ART and completed the pharmacokinetic sampling, 121 completed 6 months of follow-up, seven were lost to follow-up, four discontinued the study, and two died (both had TB/HIV coinfection). There was no documented discontinuation of ART due to medication side effects, except that zidovudine was changed to abacavir in one child who developed anemia, which required blood transfusion. From baseline values, the average aspartate aminotransferase (AST) level decreased by 2.63 and the alanine aminotransferase (ALT) increased by 2.25 at 4 weeks of ART. Of the 60 children who had viral load data at 6 months of ART, 47 (78%) had <200 copies/ml HIV RNA. The children with TB/HIV coinfection were significantly more likely than those with only HIV to have unsuppressed HIV RNA at 6 months of ART (38.9% versus 14.3%, *P* = 0.034). There were no significant differences in efavirenz *C*_max_, *C*_12h_, *C*_min_, and AUC_0–24h_ values or CYP2B6 516TT genotype frequency between the children with <200 copies/ml HIV RNA versus those with levels of ≥200 copies/ml (Table S3).

## DISCUSSION

At the population level, efavirenz mean plasma exposure and trough and middose concentrations in children with TB/HIV coinfection on first-line anti-TB therapy were similar to those in children with HIV infection without TB. The comparable PK parameters between the two populations in our study was likely due to a net inhibitory effect of 4-drug anti-TB therapy on efavirenz clearance in children, as we found higher mean efavirenz AUC_0–24h_ and lower CL/*F* on than those in patients off first-line anti-TB therapy. While our findings of comparable PK parameters in HIV-infected children with TB coinfection on anti-TB therapy and those without TB is reassuring, there was a trend toward worse HIV RNA suppression rates in the TB/HIV-coinfected participants, which was not explained by low efavirenz concentrations. Thus, a prospective clinical efficacy study that incorporates efavirenz PK parameters, as well as medication adherence and *CYP2B6* genotypes in a predictive model for virologic response, is needed to inform strategies to optimize efavirenz-based ART in children with TB/HIV coinfection.

The higher efavirenz mean concentrations on compared to off first-line anti-TB therapy among the TB/HIV-coinfected children with paired samples in our study is consistent with findings of studies among African adults with TB/HIV coinfection (21, 22). However, other studies found no significant difference in mean or median efavirenz concentrations on compared to those in patients off anti-TB therapy (23, 24). In children, previous published studies reported no difference in middose or trough efavirenz concentrations on compared to off anti-TB therapy (25, 26). Among South African children, median average efavirenz concentrations on anti-TB therapy were 1.64 mg/liter on compared to 1.96 mg/liter off anti-TB therapy and 1.7 mg/liter among controls without tuberculosis (*P* = 0.64) (25). However, the change in efavirenz concentrations varied by *CYP2B6* genotype, such that concomitant anti-TB therapy increased efavirenz concentrations 1.49-fold (95% confidence interval [CI], 1.20 to 2.01) in children with slow metabolizer genotypes but did not affect efavirenz concentrations in extensive or intermediate metabolizers (25). In another study among South African children, median efavirenz *C*_min_ was 0.83 mg/liter on and 0.86 mg/liter off anti-TB therapy (*P = *0.125) (26). While the effect of *CYP2B6* genotype was not examined in the later study, the four children with highest efavirenz concentrations had remarkably higher concentrations on compared to off anti-TB therapy (26). We found a significantly lower efavirenz CL/*F* and higher efavirenz *C*_max_, *C*_12h_, and AUC_0–24h_ values on than off anti-TB therapy in the current study. The difference in findings between our study and those of the above-mentioned South African studies (25, 26) may be due to sample size, differences in proportion of participants with *CYP2B6* 516TT genotype, and/or the higher dosages of isoniazid and rifampin used in our study. We previously found in an *in vitro* study that the inhibitory effect of isoniazid increases with increasing concentration (16). It is possible that the higher isoniazid dose (median, 10 mg/kg) used in our study, as opposed to the previously recommended 5 mg/kg, may have resulted in a larger net inhibition effect on efavirenz clearance. Given the small number of TB/HIV-coinfected children who had both anti-TB and efavirenz PK data in our study, we were not able to adequately examine the relationship between isoniazid concentrations and efavirenz PK.

We found an increase in mean efavirenz clearance after stopping anti-TB therapy in our study population, although some children had an increase, whereas others had a decrease (Fig. 2). Isoniazid is a potent mechanism-based inhibitor of CYP2A6 (16), but pyrazinamide and ethambutol have no significant inhibitory effect on the CYP and UGT enzymes involved in efavirenz metabolism (27). Efavirenz is known to induce its own metabolism over 16 weeks of efavirenz-based ART in adults especially in those with a CYP2B6*1/*1 genotype through 8-hydroxylation (28), but efavirenz had no significant additive or synergistic effect over that due to ongoing rifampin-containing therapy in TB/HIV-coinfected adults (29). Thus, our findings suggest that inhibitory effect of isoniazid may have overcome the induction effect of rifampin during coadministration of the first-line anti-TB regimen with efavirenz in some children. As previously observed, the greatest inhibition by rifampin/isoniazid-containing therapy was seen in individuals with *CYP2B6* slow metabolizer genotypes, rather than in intermediate or extensive metabolizers. We found a similar trend in the current study, but the difference did not reach statistical significance (data not shown). We do not think that the increase in efavirenz clearance following discontinuation of anti-TB therapy was due to increased age of the patients (i.e., a developmental effect), because CYP2B6 activity appears as early as the first day of life, and CYP2B6 levels and activity approach those of adults by 1 year of age (30).

**FIG 4:**
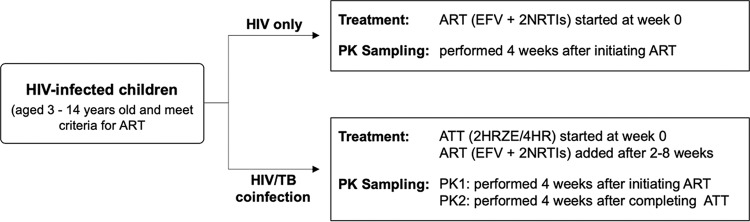
Timeline of the study design. ART, antiretroviral therapy; ATT, antituberculosis therapy; E, ethambutol; EFV, efavirenz; H, isoniazid; HIV, human immunodeficiency virus infection; NRTIs, nucleoside reverse transcriptase inhibitors; PK, pharmacokinetic; PK1, first pharmacokinetic sampling in HIV/TB infected children; PK2, second pharmacokinetic sampling in HIV/TB infected children; R, rifampin; TB, tuberculosis; Z, pyrazinamide.

Regarding virologic outcome, we found a higher rate of unsuppressed HIV RNA in the children with TB/HIV coinfection than those with only HIV infection for whom viral load data were available at 6 months of ART. However, there were no significant differences in efavirenz concentration thresholds or PK parameters between the children with suppressed and unsuppressed viral load. Several thresholds of efavirenz concentrations have been proposed for virologic response, but none of those studies included TB/HIV-coinfected patients on anti-TB therapy. Efavirenz middose (or trough) concentrations of <1 mg/liter was associated with increased risk of virological failure in HIV-infected adults who did not have TB (19, 31, 32), while concentrations above 4 mg/liter have been associated with risk of central nervous system (CNS) side effects (19, 32). Among South African children, efavirenz *C*_12h_ of 1.12 mg/liter, concentration at 24 h postdose (*C*_24h_) of 0.65 mg/liter, and AUC_0–24h_ of 28 mg · h/liter were found to be predictive of increased risk of unsuppressed viral load (20). In contrast, one study among children found no correlation between efavirenz concentrations and viral load decrease at 3 months (33). In addition, among 15 South African children with TB/HIV coinfection, 60% and 53% of the participants had a *C*_min_ of <1 mg/liter on and off TB treatment, respectively, but the virologic suppression rate after 6 months of ART was 80% (26). We found no clear relationship between proposed PK parameter thresholds for clinical outcome and virologic suppression. A key limitation of our study is that PK sampling was performed only once, at 4 weeks of ART, and poor adherence after the sampling visit could have influenced the risk of virologic response at 6 months of ART. In addition, only 57% of our participants had 6 months of viral load data; we thus did not have enough data to explore a relationship between PK parameters and virologic response. We found a significant difference in PK parameters between the children with HIV infection and those with TB/HIV coinfection after stopping the anti-TB therapy. However, given the difference in timing of PK sampling (Fig. 4), it is not known whether increased efavirenz clearance off anti-TB therapy could have occurred by a mechanism unrelated to TB treatment or discontinuation. We have initiated a follow-up study that would compare 6 and 12 months virologic response in HIV-infected versus TB/HIV-coinfected children on efavirenz-based therapy, while accounting for other factors such as medication adherence, *CYP2B6* 516G→T genotype and efavirenz middose concentrations. Finally, given that the sampling interval was only up to 24 h postdose, the reported *t*_1/2_ is an estimation.

In conclusion, we found that first-line anti-TB therapy in children led to decreased efavirenz clearance and increased efavirenz plasma exposure in TB/HIV-coinfected patients. In addition, the children with TB/HIV coinfection generally had lower body weights and weight-for-age *Z* scores. Thus, they received a higher efavirenz dosage when the same weight-band dosing recommendation was used. The net inhibitory effect of first-line anti-TB therapy on efavirenz clearance and the comparable efavirenz PK parameters in children with TB/HIV coinfection on anti-TB therapy and those with HIV infection in our study do not support a modified weight-band dosing of efavirenz in children with TB/HIV coinfection during anti-TB therapy. However, further clinical studies are warranted to determine whether TB coinfection and concurrent anti-TB therapy adversely affect long-term virologic response in TB/HIV-coinfected children treated with efavirenz-based therapy.

## MATERIALS AND METHODS

### Study design.

A two-arm parallel assignment efavirenz PK study (Fig. 4) was performed between October 2012 and November 2017 at the Komfo Anokye Teaching Hospital (KATH), Kumasi, Ghana. ART-naive HIV-infected children aged 3 to 14 years old, with or without active TB and eligible to initiate efavirenz-based ART, were recruited into the study. Children with opportunistic infections other than TB or who had acute illness other than malnutrition or whose parents declined to sign written consent were excluded. The Institutional Review Board (IRB) of KATH (Ghana), as well as those of the principal investigator institutions (Lifespan Hospitals, Rhode Island, and University of Florida) reviewed and approved the study. All parents or guardians of study participants provided written informed consent. The study was registered with ClinicalTrials.govNCT01704144.

At enrollment, complete medical history, physical examination, and nutritional status assessments were performed, and relevant data were collected using standardized forms. Baseline measurements prior to initiation of ART included complete blood count (CBC), blood urea nitrogen, serum creatinine, and liver function tests (LFTs), as well as CD4 cell count determination and plasma HIV-1 RNA levels. Study participants were evaluated after 2 weeks of ART and then monthly to assess for adverse events and clinical response to therapy. Liver function tests were repeated at week 4 of ART, and CD4 cell count and HIV-1 plasma RNA levels were repeated after 12 and 24 weeks of ART. However, as shown in Table 1, some participants did not complete all of the planned laboratory testing due to missed sample collection, failed laboratory testing, lack of reagents, or broken equipment at the study site during the study period.

### Treatment regimens.

The antiretroviral regimen consisted of efavirenz plus two nucleoside reverse transcriptase inhibitors (NRTIs). The daily efavirenz dose was based on the following weight-band dosing recommendation according to WHO and Ghana National HIV treatment guidelines: 10 to 15 kg (200 mg), 15 to 25 kg (300 mg), 25 to 40 kg (400 mg), and >40 kg (600 mg). Additional weight bands that were in used at the beginning of the study but were later phased out by the Ghana National AIDS Control Program were 15 to 20 kg (250 mg) and 25 to 33 kg (350 mg). The anti-TB regimen consisted of isoniazid, rifampin, pyrazinamide, and ethambutol daily for 2 months, and then isoniazid and rifampin daily for 4 months. The medications were dosed according to WHO guidelines for using available dispersible fixed-dose combination (FDC) TB medicines for children (34).

### Pharmacokinetic sampling and analysis.

Pharmacokinetic blood sampling was performed after at least 4 weeks of ART in both arms, and a second sampling was performed after at least 4 weeks of stopping the anti-TB treatment in the TB/HIV-coinfected group. On the day of sampling, medications were administered after an overnight fast. Medications were either swallowed or dispersed in water in a plastic cup and ingested. A light standard breakfast was provided 30 min after dosing. Once the 2-h sample was obtained, children were allowed to eat without restrictions. Blood samples were collected at times 0, 2, 8, 12, and 24 h postdose for determination of efavirenz concentrations. The samples collected in EDTA-coated tubes were centrifuged within 30 min at 3,000 × *g* for 10 min. Plasma was stored at −80°C until shipment on dry ice to University of Cape Town (Cape Town, South Africa) for drug concentration assays. Efavirenz concentrations in plasma were measured using validated liquid chromatography with tandem mass spectrometry (LC-MS/MS) methods (35). Throughout the course of analysis, the accuracy of the assay was shown to be 98.8%, with an associated precision (percent coefficient of variation [CV%]) of 6.4 across all three quality control concentrations. The maximum or peak concentration (*C*_max_), time to *C*_max_ (*T*_max_), and minimum concentration (*C*_min_) were determined by inspection of the plasma concentration-time graphs for efavirenz. The calculations of area under the curve from time zero to 24 h (AUC_0–24h_), estimated apparent oral clearance (CL/*F*), and volume of distribution (*V*/*F*) were performed using noncompartmental analysis (Phoenix Software; Pharsight Corporation, Mountain View, CA).

### Genotyping of human allelic variants.

Genotyping for *CYP2B6* 516G→T single nucleotide polymorphisms (SNPs) was performed by TaqMan allelic discrimination on a QuantStudio 12K Flex system (Life Technologies, Foster City, CA) at University of Florida Center for Pharmacogenomics and Precision Medicine. Other functional SNPs in *CYP2A6, CYP3A4/5*, and *ABCB1* were also performed, but these SNPs were not included in the current analysis given the lack of significant effect in exploratory analysis (data not shown).

### Statistical analysis.

Statistical analyses were performed using SAS 9.4 software (SAS Institute Inc, Cary, NC). Weight-for-age *Z* score (WAZ), height-for-age *Z* score (HAZ), and body mass index (BMI) for age were calculated based on the United States National Center for Health Statistics (NCHS) reference median values, using statistical macros for children aged <5 years old and 5 to 19 years old provided by the WHO (36). Bivariate analyses of association between patient factors and efavirenz PK parameters were performed using the Wilcoxon rank-sum test for continuous variables and the Fisher exact test for categorical variables. The signed-rank test was applied to compare within-group change of PK parameters for the TB/HIV coinfection group on and off TB treatment. Multivariate regression was used to explore the joint effect of demographics and clinical variables on the PK parameters. The stepwise variable selection was used to select the predictors of efavirenz PK parameters. While an effect was added to or removed from the model based on the significance level of the *F* statistic, the corrected Akaike information criterion (AICC) was used to stop the selection process. For all analyses, a *P* value of <0.05 was considered significant.

## Supplementary Material

Supplemental file 1
