# Supplementary material for: Effect of Rifampin-Isoniazid-Containing Antituberculosis Therapy on Efavirenz Pharmacokinetics in HIV-Infected Children 3 to 14 Years Old
Source: Antimicrob Agents Chemother. 2018 Dec 21;63(1):e01657-18. doi: 10.1128/AAC.01657-18 (PMC6325194; doi:10.1128/AAC.01657-18)
Supplement: Supplemental file 1 [file 216eccc982f6d3f42f03ae10abc7e0cd_AAC.01657-18-s0001.pdf]

Supplement Table 1. Median (interquartile range) pharmacokinetic parameter estimates of efavirenz in Ghanaian HIV-infected children with and without tuberculosis

**Parameter**	**All (N=105)#**	**HIV (N = 62)**	**TB/HIV (N = 43)**	**P value**
T_{max} (hr)	2.1 (2.0 – 2.4)	2.1 (2.0 – 2.3)	2.1 (2.0 – 8.0)	0.128
C_{max} (mg/L)	3.8 (2.8 – 5.4)	4.2 (3.0 – 5.4)	3.5 (2.6 – 5.1)	0.166
C_{12h} (mg/L)	2.0 (1.3 – 3.1)	2.1 (1.3 – 3.6)	1.8 (1.2 – 3.1)	0.513
C_{min} (mg/L)	1.7 (1.0 – 2.8)	1.8 (1.2 – 3.1)	1.4 (0.8 – 2.8)	0.199
AUC_{0-24h} (mg *hr/L)	52.9 (35.1 – 84.8)	56.0 (40.1 – 84.9)	47.3 (33.8 – 79.2)	0.262
CL/F (L/hr)	5.5 (3.1 – 7.5)	5.5 (3.2 – 7.6)	5.9 (3.0 – 7.5)	0.843
V/F (L)	135.9 (94.9 – 203.7)	150.8 (98.1 – 218.1)	129.3 (91.0 – 196.8)	0.486
$t_{1/2}$ (hr)	20.1 (13.3 – 28.6)	21.3 (15.7 – 28.6)	16.2 (11.0 – 29.3)	0.181

#T_{max} could not be estimated in one participant with HIV infection, AUC_{0-24h} = total area under the curve from time 0-24 hours, C_{12h} = concentration at 12 hour post dose, C_{max} = peak concentration, C_{min} = minimum concentration, CL/F = apparent oral clearance, $t_{1/2}$ = half-life, T_{max} = time to C_{max}, V/F = apparent volume of distribution.

Supplement Table 2. Proportion of HIV-infected children with and without tuberculosis (TB) coinfection who had pharmacokinetic parameters outside various proposed efavirenz therapeutic thresholds

**Parameter**	**All (N=105)**	**HIV (N=62)**	**TB/HIV (N=43)**	**P value**
C_{12h} < 1 mg/L	10 (9.5%)	4 (6.5%)	6 (14.0%)	0.310
C_{12h} < 1.12 mg/L	18 (17.1%)	10 (16.1%)	8 (18.6%)	0.741
C_{min} < 1 mg/L	24 (22.9%)	9 (14.5%)	15 (34.9%)	0.015
C_{min} < 0.65 mg/L	14 (13.3%)	7 (11.3%)	7 (16.3%)	0.460
C_{12h} > 4 mg/L	22 (21.0%)	13 (21%)	9 (20.9%)	0.996
C_{min} > 4 mg/L	16 (15.2%)	9 (14.5%)	7 (16.3%)	0.805
AUC_{0-24h} < 28 mg.h/L	12 (11.4%)	6 (9.7%)	6 (14.0%)	0.498

Numbers (percent) are reported for categorical data, AUC_{0-24h} = total area under the curve from time 0-24 hours, C_{12h} = concentration at 12 hour post dose (mid-dose), C_{min} = minimum concentration.

Supplement Table 3. Differences in median (interquartile range efavirenz pharmacokinetic parameters and characteristics of patients with suppressed and unsuppressed HIV RNA at 6 months

Parameter	HIV RNA < 200 c/mL (N =47)	HIV RNA ≥ 200 c/mL (N =13)	P-value
C_{max} (mg/L)	3.6 (2.8 – 5.4)	4.0 (3.6 – 5.4)	0.532
C_{12h} (mg/L)	1.9 (1.2 – 2.7)	2.2 (1.9 – 3.6)	0.227
C_{min} (mg/L)	1.6 (1.1 – 2.5)	1.9 (1.0 – 2.8)	0.789
$AUC_{0\text{-}24h}$ (mg *hr/L)	52.9 (33.8 – 72.5)	55.8 (46.9 – 84.8)	0.328
CL/F (L/hr)	5.6 (3.8 – 8.4)	4.3 (3.5 – 6.4)	0.255
V/F (L)	159.1 (101.3 – 229.2)	135.9 (100 – 187.4)	0.476
TB/HIV coinfection			0.034
No	36 (85.7%)	6 (14.3%)	
Yes	11 (61.1%)	7 (38.9%)	
Sex			1.000
Female	17 (77.3%)	5 (22.7%)	
Male	30 (78.9%)	8 (21.1%)	
Efavirenz C_{min}			0.890

< 1 mg/L	8 (80.0%)	2 (20.0%)	
1 – 4 mg/L	31 (75.6%)	10 (24.4%)	
> 4 mg/L	8 (88.9%)	1 (11.1%)	
Efavirenz C_{12h}			0.890
< 1 mg/L	6 (85.7%)	1 (14.3%)	
1 – 4 mg/L	33 (78.6%)	9 (21.4%)	
> 4 mg/L	8 (72.7%)	3 (27.3%)	
CYP2B6 516G>T genotype			0.294
GG/GT	34/42 (81.0%)	8/42 (19.0%)	
TT	10/15 (66.7%)	5/15 (33.3%)	

Numbers (percent) are reported for categorical data, AUC_{0-24h} = total area under the curve from time 0-24 hours, C_{12h} = concentration at 12 hour post dose (mid-dose), C_{max} = peak concentration, C_{min} = minimum concentration, CL/F = apparent oral clearance, T_{max} = time to C_{max}, V/F = apparent volume of distribution.
